# The Landscape of SPP1 ^+^ Macrophages Across Tissues and Diseases: A Comprehensive Review

**DOI:** 10.1111/imm.13952

**Published:** 2025-05-21

**Authors:** Alessandro Palma

**Affiliations:** ^1^ Department of Biology and Biotechnologies “Charles Darwin” Sapienza University of Rome Rome Italy

**Keywords:** cancer, fibrosis, immunosuppression, innate immunity, osteopontin, SPP1 macrophages, tumour‐associated macrophages

## Abstract

Macrophages play a critical role in shaping the immune landscape of various diseases, with secreted phosphoprotein 1 (SPP1)‐expressing macrophages emerging as a distinct subset implicated in both cancerous and non‐cancerous conditions. Leveraging recent advances in single‐cell RNA sequencing, numerous studies have identified SPP1^+^ macrophages across diverse pathological contexts, shedding light on their functional heterogeneity. In cancer, SPP1^+^ tumour‐associated macrophages contribute to tumour growth, angiogenesis, and immune evasion, often interacting with T cells and stromal components to sustain an immunosuppressive microenvironment. Conversely, in non‐cancerous diseases, these macrophages exhibit both profibrotic and disease‐promoting properties, depending on the tissue context. This review provides a comprehensive synthesis of the latest findings on SPP1^+^ macrophages, highlighting their roles in tumour progression, immune suppression, tissue remodelling, and fibrosis. By comparing their shared traits and tissue‐specific differences, we explore how SPP1^+^ macrophages adapt to distinct microenvironments and influence disease progression. Understanding their conserved and context‐dependent functions may open new avenues for therapeutic targeting.

## Graphical abstract

SPP1^+^ macrophages represent a conserved, disease‐associated population present across cancerous and non‐cancerous conditions, involved in immunosuppression, fibrosis, lipid metabolism, phagocytosis, and other cellular processes. This review highlights their shared molecular programmes across tissues, their interactions with stromal and immune cells, and their context‐specific functional diversity beyond classical M1/M2 polarisation.

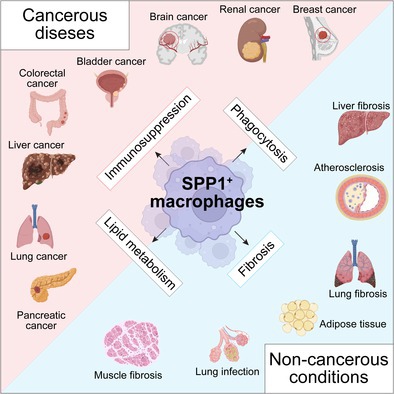

**Abbreviations**
APOC1: apolipoprotein C1
APOC2: apolipoprotein C2
APOE: apolipoprotein E
ARG1: arginase 1
ARG2: arginase 2
BALF: bronchoalveolar lavage fluid
CAF: cancer‐associated fibroblast
CCL5: chemokine (C—C motif) ligand 5
CCR2: C—C chemokine receptor type 2
CD9: cluster of differentiation 9
CD36: cluster of differentiation 36
CD44: cluster of differentiation 44
CD68: cluster of differentiation 68
CD163: cluster of differentiation 163
CHI3L1: chitinase‐3‐like protein 1
CHIT1: chitinase 1
CLEC5A: C‐type lectin domain family 5 member A
CNS: central nervous system
COL11A1: collagen type XI alpha 1 chain
CRC: colorectal cancer
CSFR1: colony stimulating factor 1 receptor
CTSB: cathepsin B
CTSD: cathepsin D
CXCL4: chemokine (C‐X‐C Motif) ligand 4
CXCL9: chemokine (C‐X‐C Motif) ligand 9
CXCL10: chemokine (C‐X‐C Motif) ligand 10
DAM: damage‐associated microglia
ECM: extracellular matrix
FABP4: fatty acid binding protein 4
FABP5: fatty acid binding protein 5
FAP: fibroblast activation protein alpha
FBP1: fructose‐bisphosphatase 1
FTL: ferritin light chain
FN1: fibronectin 1
GPR183: G‐protein coupled receptor 183
HER2: human epidermal growth factor receptor 2
HR: hormone receptor
ICC: intrahepatic cholangiocarcinoma
IFN©: interferon gamma
IGF1: insulin growth factor 1
IL1B: interleukin 1 beta
IL1RN: interleukin‐1 receptor antagonist
IL6: interleukin 6
IPF: idiopathic pulmonary fibrosis
iPSC: induced pluripotent stem cells
LAM: lipid‐associated macrophage
LAPTM5: lysosomal protein transmembrane 5
LGALS3: galectin 3
LGALS9: galectin 9
LGMN: legumain
LMAM: lipid metastasis‐associated macrophage
LPL: lipoprotein lipase
LRRC15: leucine rich repeat containing 15
LYVE1: lymphatic vessel endothelial hyaluronan receptor 1
GPNMB: glycoprotein Nmb
MAP K14: mitogen‐activated protein kinase 14
MARCO: macrophage receptor with collagenous structure
MIF: macrophage migration inhibitory factor
MMP9: matrix metalloproteinase 9
MoAM: monocyte‐derived alveolar macrophage
MT1G: metallothionein 1G
MRC1: mannose receptor C‐type 1
NLRP3: NLR family pyrin domain containing 3
OLR1: oxidised Low Density Lipoprotein Receptor 1
PDGFRα: platelet derived growth factor receptor alpha
PD‐L1: programmed death ligand 1
PF4: platelet factor 4
PPAR©: peroxisome proliferator activated receptor gamma
RNASE1: ribonuclease A family member 1, pancreatic
SAM: scar‐associated macrophage
S100A11: S100 calcium binding protein A11
S100P: S100 calcium binding protein P
STMN1: stathmin 1
SYNGR1: synaptogyrin 1
TGF‐β: transforming growth factor beta
TNBC: triple‐negative breast cancer
TNFα: tumour necrosis factor alpha
TNFSF12: TNF superfamily member 12
Tregs: regulatory T‐cells
TREM2: triggering receptor expressed on myeloid cells 2
VEGFA: vascular endothelial growth factor A
VCAN: versican

## 1 Introduction

Macrophages are essential sentinels of tissue homeostasis, dynamically responding to microenvironmental changes to maintain balance [1]. Their functions extend beyond immune surveillance, as they clear apoptotic cells, eliminate cancerous and damaged cells, and facilitate tissue regeneration by secreting trophic factors. Additionally, they orchestrate immune responses to pathogens, positioning themselves at the crossroads of immunity and tissue remodelling [2].

In the context of cancer, macrophages adopt a dual role, acting both as defenders against tumour cells and as facilitators of tumour progression. These tumour‐associated macrophages (TAMs) are among the most abundant immune cells in the tumour microenvironment (TME), where they shape tumour growth, support metastasis, and contribute to immune evasion [3]. Many TAMs originate from circulating monocytes recruited by tumour‐secreted signals, undergoing a functional reprogramming that skews their activity toward a pro‐tumoral phenotype [4, 5, 6].

Macrophages, however, are far from a uniform population [7]. Their functional diversity has spurred extensive classification efforts, initially based on a simplified polarisation model distinguishing pro‐inflammatory (M1) and anti‐inflammatory (M2) states. This binary view has since evolved, with the recognition that macrophages exist across a continuum of activation states, influenced by their tissue of origin, residency status, and microenvironmental cues [8]. The advent of single‐cell technologies has further expanded our understanding, revealing distinct macrophage subsets with specialised functions.

Among this diverse cell population, SPP1^+^ macrophages, characterised by high expression of secreted phosphoprotein 1 (SPP1), which encodes Osteopontin (OPN), have garnered significant attention. Because the majority of single‐cell transcriptomic studies have so far focused on cancer, macrophage subpopulations expressing high levels of *SPP1* have been predominantly identified and characterised in tumours (see “SPP1 Macrophages in Cancer” chapter). However, growing evidence now suggests that these macrophages are not restricted to cancer; they also play crucial roles in non‐cancerous diseases, highlighting their broader relevance in pathology. This raises fundamental questions about their function, plasticity, and contribution to disease progression.

In this review, we explore the biology of SPP1^+^ macrophages, both in cancer and non‐cancerous conditions, to provide a comprehensive synthesis of their roles in disease. We begin with an overview of SPP1's functions, followed by an in‐depth examination of SPP1^+^ macrophages across different pathological contexts. Finally, we discuss their shared traits, tissue‐specific differences, and the current limitations in our understanding, while identifying key areas for future research. By bringing together these insights, we aim to shed light on the significance of SPP1^+^ macrophages and their potential as therapeutic targets.

## 2 Osteopontin

Osteopontin (OPN) is a glycosylated protein encoded by the *SPP1* gene. It was first discovered as a phosphoprotein secreted by transformed cells [9], and then cloned as a bone‐specific sialoprotein from the rat osteosarcoma phage λgt11 library (from which the name “Osteopontin”) [10].

**FIGURE 1:**
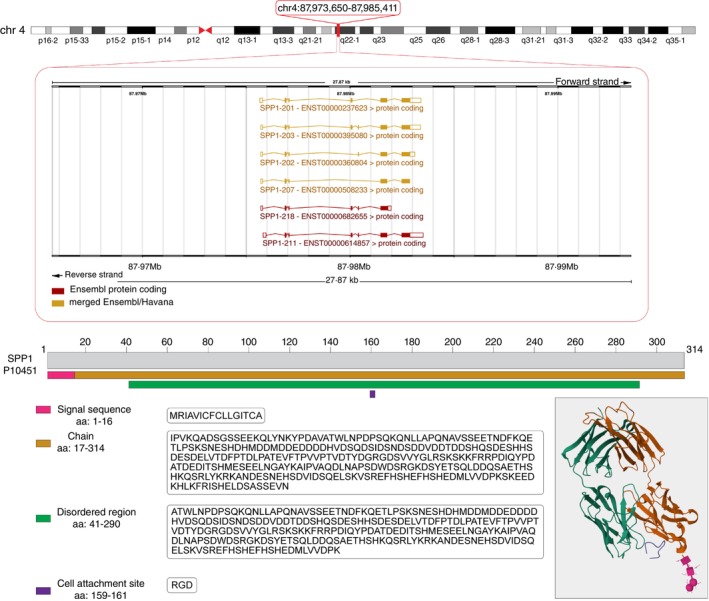
Human SPP1 genomic and proteomic features. Upper panel: SPP1 genome locus (human genome hg38) along with the protein‐coding transcript isoforms. Lower panel: Uniprot information of the main isoform of SPP1 protein (P10451) showing protein structural features, domains, and Protein Data Bank structure.

Based on the current human Ensemble annotation (GRCh38.p14), the *SPP1* gene is located on the forward strand of the long arm of chromosome 4. It encodes 26 distinct transcript isoforms, six of which form proteins with a length that ranges from 192 to 314 amino acids (Figure 1).

Two types of SPP1 proteins have been studied so far: a secreted SPP1 (also known as sOPN) and an intracellular SPP1 (also known as iOPN), with distinct functions based on their different cellular localisation [11]. While iOPN is involved in cytoskeletal rearrangement and in signal transduction pathways, participating also in the negative regulation of myelopoiesis [12], sOPN is rather involved in cell adhesion and migration through interactions with integrins and other receptors like CD44 [11]. However, iOPN is still able to bind to receptors like integrins and CD44 and plays a role in migration, cell fusion, and resorption in osteoclasts [13]. Other functions of the SPP1 protein include bone remodelling, anchoring osteoclasts to the bone mineral matrix, regulation of the innate and adaptive immune systems, cell differentiation, cytokine production, and cancer progression, among others (reviewed in [14]).

## 3 An Overview of Macrophage Markers in Physiology and Cancer

Macrophages are a remarkably diverse and adaptable group of immune cells, encompassing both long‐lived tissue‐resident populations and monocyte‐derived infiltrating cells. Historically, their functional states have been categorised into two broad classes, M1 (pro‐inflammatory) and M2 (anti‐inflammatory), based on their responses to environmental cues. While this M1/M2 framework has provided a useful starting point, it is now widely recognised as an oversimplification. In reality, macrophage activation spans a dynamic and continuous spectrum of phenotypes, influenced by a complex array of signals in the tissue microenvironment [8].

Despite these advances, the M1/M2 classification remains a valuable conceptual tool, particularly in experimental and clinical studies that require quantifiable endpoints. For instance, M1‐like macrophages are typically activated by Toll‐like receptor ligands, such as bacterial lipopolysaccharide (LPS), as well as Th1‐type cytokines including tumour necrosis factor‐alpha (TNFα), interferon‐gamma (IFNγ), and colony‐stimulating factor 2 (CSF2). These cells are associated with antimicrobial and tumoricidal activities. In contrast, M2‐like macrophages are induced by Th2 cytokines such as IL‐4, IL‐10, IL‐13, and transforming growth factor‐beta (TGFβ), and are more commonly linked to tissue repair, fibrosis, and immunosuppression [15].

In the context of cancer, tumour‐associated macrophages (TAMs) are a key component of the tumour microenvironment (TME), where they often adopt an M2‐like, pro‐tumorigenic phenotype, opposed to the antitumoral activities of the M1‐like macrophages [16, 17, 18]. M2 TAMs can promote tumour progression by secreting proteolytic enzymes, angiogenic growth factors, and immune checkpoint molecules, which collectively support tumour growth, invasion, and immune evasion [19]. Conversely, TAMs with M1‐like features have been shown to exert anti‐tumoral effects, although such populations are generally less abundant within established tumours [20]. A non‐exhaustive list of common M1‐like and M2‐like TAM markers is provided in Table 1 and extensively reviewed in [19, 37, 38].

**TABLE 1:** Tumour‐associated macrophage markers.

Marker	Name	M1/M2	Reference
CD68	Cluster of differentiation 68	Both	[21, 22, 23]
CD163	Cluster of differentiation 163	Both (generally an M2‐like marker)	[24, 25, 26]
CD206	Cluster of differentiation 206	M2	[27, 28]
FOLR2	Folate receptor beta	M2	[29, 30]
NOS2	Nitric oxide synthase	M1	[31, 32]
CD204	Cluster of differentiation 204	M2	[33, 34]
STAB1	Stabilin 1	M2	[35, 36]

*Note*: Commonly used markers to distinguish between pro‐ and anti‐tumoural TAMs in different cancers.

It is worth noting that while some markers are widely accepted as indicative of either M1 or M2 polarisation, others show context‐dependent expression that varies by tissue, organ, or disease state. Additionally, certain markers have been primarily studied in specific pathological settings or may differ based on macrophage identity, for example, between microglia and circulating monocyte‐derived macrophages.

The SPP1 marker has been detected in diverse macrophage populations and is frequently associated with cells exhibiting an M2‐like polarisation profile, as explored in greater detail in the subsequent chapters of this review.

## 4 SPP1 Macrophages in Cancer

### 4.1 Brain Cancer

Macrophages in brain cancer represent the most abundant immune cells in the tumour microenvironment [39]. They are recruited from circulating monocytes to tumours, and they exert important functions supporting tumour proliferation, regulating immunosuppression, and favouring tumour resistance to therapy [5, 40].

In recent years, various studies on TAMs and microglia—the brain‐resident macrophages—within the brain and central nervous system discovered previously unrecognised or understudied populations of TAMs and microglia expressing high levels of *SPP1*, in both human and murine models [41]. In particular, this macrophage population is often predominantly present in tumour tissues compared to healthy ones. Indeed, an increase in macrophages expressing high levels of *Spp1, ApoE, Apoc1, Lgals3*, and *Gpnmb* has been documented in glioma models, with high‐grade gliomas exhibiting a greater abundance of these macrophages compared to low‐grade gliomas [42]. In this context, SPP1^+^ macrophages emerge as key immunosuppressive actors during glioma progression, being implicated in tumour progression and invasion, partly through their association with extracellular matrix remodelling factors like cathepsins, shaping an immunosuppressive tumour microenvironment, and correlating with decreased overall survival. Moreover, single‐cell RNA sequencing analyses of glioma samples have identified a TAM subset characterised by high *SPP1* expression, alongside other gene markers such as *FTL*, *LAPTM5*, *S100A11*, *APOC1*, and *APOC2* [43]. This subset is thought to suppress T‐cell activity through SPP1–CD44 and LGALS9–CD44 interactions, highlighting its potential immunosuppressive role in the glioma microenvironment [43].

**FIGURE 2:**
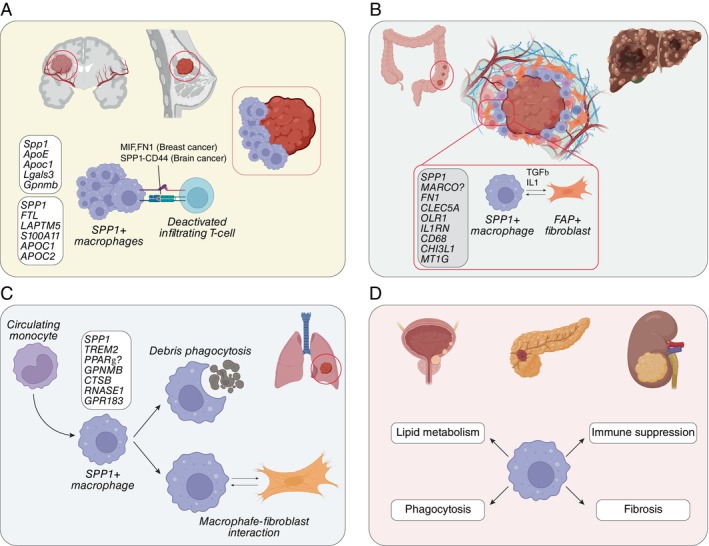
SPP1^+^ macrophages in cancer. (A) In brain and breast cancer, SPP1 macrophages are localised at the boundaries of the tumour mass and communicate with infiltrating T‐cells to create an immunosuppressive environment. (B) In colon and liver cancer, SPP1 macrophages interact with FAP‐positive fibroblasts. (C) In lung cancer, monocyte‐derived SPP1^+^ macrophages recruited by the tumour cells phagocytize tumoral cell debris and interact with fibroblasts, shaping the tumour microenvironment. (D) Representation of the main roles of SPP1^+^ macrophage in other tumour types, including bladder, pancreatic, and renal cancers.

TAMs are well known to promote glioma cell survival and angiogenesis, and SPP1^+^ TAMs have been implicated in tumour progression via their interaction with β1 integrin [44]. Additionally, inferred myeloid–glioma interactions suggest that the SPP1–CD44 axis plays a role in promoting cancer stemness [45, 46]. However, conflicting findings have emerged, as some studies indicate that the absence of SPP1 correlates with more aggressive tumour behaviour and reduced overall survival in mouse models of glioma [47] (Figure 2A).

These discrepancies may reflect species‐specific differences or suggest a context‐dependent role of SPP1^+^ macrophages. While these cells may initially arise as a physiological response to tumour formation, their presence could paradoxically contribute to disease progression by fostering an immunosuppressive and pro‐tumorigenic microenvironment.

Altogether, these findings position SPP1^+^ TAMs as critical regulators of the glioma immune landscape. Their association with immunosuppressive signalling pathways (e.g., SPP1–CD44) and influence on tumour invasiveness and stemness suggest a multifaceted role in tumour progression. At the same time, conflicting evidence regarding their impact on survival underscores the complexity and potential context‐dependence of their function, possibly influenced by tumour stage, spatial distribution, or interspecies differences. Further research is needed to clarify the precise mechanisms by which SPP1^+^ TAMs influence brain tumour pathophysiology and whether targeting this pathway could offer therapeutic potential.

### 4.2 Breast Cancer

As in many other tumour types, breast cancer is characterised by a strong infiltration of macrophages, among which the SPP1^+^ subset is gaining increasing attention. The immune landscape of breast cancer plays a crucial role in shaping disease progression and prognosis. In breast cancer, a high infiltration of lymphocytes is associated with better clinical outcomes, while lower immune cell infiltration correlates with poorer overall survival [48]. Within this dynamic context, macrophages act as key regulators of immune function, often tipping the balance in favour of tumour progression and poor prognosis [6].

Recent studies have unveiled a striking role for SPP1^+^ macrophages in HR+ breast cancer, where they establish interactions with tumour‐infiltrating lymphocytes and actively suppress their function. This immunosuppressive effect is mediated through SPP1, MIF, and FN1 signalling, potentially explaining the adverse prognosis associated with lower T‐lymphocyte infiltration in this subtype [49] (Figure 2A). Unlike their angiogenic role observed in other tumour contexts, SPP1^+^ macrophages in breast cancer appear to drive fibrosis and extracellular matrix remodelling, processes that may further fuel tumour progression.

The clinical significance of SPP1 has been further highlighted in recurrent breast cancers, where its levels are markedly elevated, primarily due to macrophages, though endothelial and other stromal cells also contribute [50]. Notably, experimental depletion of monocyte‐derived macrophages using clodronate liposomes, or direct inhibition of Osteopontin, has been shown to reduce tumour recurrence and improve anti‐tumour immunity in preclinical models, highlighting SPP1 as a potential therapeutic target to prolong relapse‐free survival [50].

Beyond their individual impact, SPP1^+^ TAMs have been described with varying marker profiles across studies [51], which may suggest distinct subpopulations. While this heterogeneity likely reflects context‐dependent expression patterns rather than discrete subsets, it may nonetheless hold clinical relevance, as targeting specific macrophage states could offer therapeutic opportunities in breast cancer [52]. Furthermore, the precise role of these cells, particularly their interactions with tumour cells and other components of the tumour microenvironment, remains an open question.

SPP1^+^ TAMs in breast cancer are emerging as pivotal modulators of the tumour microenvironment. Their role extends beyond immune suppression to include extracellular matrix remodelling and fibrosis, processes that may collectively drive tumour progression and recurrence. The correlation between SPP1 expression, poor lymphocyte infiltration, and disease relapse underscores their potential as both prognostic biomarkers and therapeutic targets. While evidence points to functional heterogeneity among SPP1^+^ macrophages, this likely reflects context‐driven plasticity rather than distinct lineages.

### 4.3 Colorectal Cancer

The tumour microenvironment in colorectal cancer (CRC) is a complex ecosystem composed of endothelial cells, fibroblasts, stromal cells, and immune infiltrates, all of which interact to influence disease progression. Within this network, TAMs play a crucial role in promoting metastasis [53]. Among them, SPP1^+^ TAMs have emerged as key players, with mounting evidence pointing to their involvement in tumour development, immune evasion, and resistance to therapy.

Recent studies reveal that CRC patients exhibit a higher abundance of SPP1^+^ TAMs, which frequently co‐express the scavenger receptor *MARCO* and engage in crosstalk with FAP+ fibroblasts (Figure 2B). This interaction forms an immunosuppressive network that restricts T‐cell infiltration, thereby weakening anti‐tumour immune responses [54]. The communication between these cell types is likely mediated by cytokines such as TGF‐β and IL‐1 family members, which not only suppress immune activation but also contribute to the reduced therapeutic efficacy of anti‐PD‐L1 treatments.

The pro‐tumorigenic role of SPP1^+^ TAMs is further underscored by their distinct gene signature, which includes *SPP1*, *FN1*, *CLEC5A*, *OLR1*, and *IL1RN*, a profile that has been strongly associated with metastasis and angiogenesis [55]. Their ability to remodel the extracellular matrix and support vascularisation may be key mechanisms underlying their metastatic potential.

Interestingly, the localisation of SPP1^+^ TAMs is not confined to metastatic sites. A recent report identified a subset of TAMs, characterised by high expression of *SPP1*, *CD68*, *CHI3L1*, and *MT1G*, preferentially residing within hypoxic tumour regions, suggesting a role in adaptation to metabolic stress [56]. This study also highlights the presence of *MARCO*‐expressing TAMs that appear distinct from the SPP1^+^ population, raising new questions about macrophage heterogeneity in CRC.

Beyond their interactions with T‐cells and fibroblasts, SPP1^+^ TAMs expressing angiogenic, matrix remodelling, and immunosuppressive markers like *Nlrp3*, *Tnf*, *Vegfa*, and *Arg1*, also engage in cross‐talk with enteric glial cells via IL1R/IL6 signalling, further influencing tumour behaviour [57]. Specifically, enteric glial cells within the tumour secrete IL‐6, which induces infiltrating monocytes to differentiate into SPP1‐expressing TAMs. This phenomenon may have important implications in cancer, as enteric glial cells are particularly abundant in CRC, and healthy enteric glial cells have been shown to induce a macrophage phenotype distinct from the SPP1^+^ subset, further highlighting the pro‐tumorigenic role of SPP1^+^ TAMs. Notably, this study describes SPP1^+^ TAMs as a terminally differentiated macrophage subset originating from monocytes. This result contrasts with reports from other tissues, where SPP1^+^ macrophages have been proposed as an intermediate, hybrid cell population arrested along a differentiation trajectory [58]. Such discrepancies may reflect methodological differences, including the use of pseudotime trajectory inference versus lineage tracing, as well as tissue‐specific influences and challenges in definitively tracing macrophage ontogeny. These contrasting interpretations underscore the complexity of defining SPP1^+^ TAM identity and function across cancer types and highlight the importance of context‐specific investigations.

Collectively, the available studies underscore the multifaceted pro‐tumorigenic role of SPP1^+^ TAMs in CRC. These macrophages contribute to metastasis, immune suppression, and therapy resistance through a combination of immunomodulatory signalling, extracellular matrix remodelling, and angiogenesis. Their interactions with fibroblasts, T cells, and enteric glial cells form complex immunosuppressive and tumour‐supportive networks, particularly under hypoxic conditions. Moreover, the apparent heterogeneity in SPP1^+^ TAMs, ranging from terminally differentiated subsets to potentially intermediate phenotypes, not only highlights the need for further investigation into their ontogeny and functional plasticity, but also positions them as potential targets for therapeutic intervention in CRC.

### 4.4 Head and Neck Cancer

Head and neck squamous cell carcinoma (HNSCC) is among the most prevalent cancers worldwide, characterised by a highly complex and heterogeneous tumour microenvironment that plays a crucial role in both disease progression and treatment response. Within this dynamic landscape, innate immune cells, particularly macrophages, have gained increasing attention, as emerging studies continue to unveil novel phenotypes and gene signatures defining their functional diversity.

Among these, SPP1^+^ TAMs have recently been identified as a key immune population in HNSCC. These cells, marked by high expression of the pan‐macrophage marker *CD68* and *SPP1*, have been strongly correlated with poor prognosis and cancer progression [59]. Their abundance within tumours is associated with shorter overall survival, mirroring findings in other malignancies where SPP1^+^ macrophages contribute to tumour aggressiveness.

Functionally, SPP1^+^ TAMs in HNSCC exhibit an M2‐like, immunosuppressive phenotype, characterised by a pro‐angiogenic and ECM‐remodelling transcriptional profile [59]. However, some reports have shown that this macrophage population can also acquire a hybrid phenotype, displaying functional diversity that does not conform to the traditional M1/M2 polarisation paradigm [60]. These features, including an M2‐like immunosuppressive phenotype or a non‐canonical polarisation state, may not only support tumour growth and invasion but also suggest a pivotal role in reshaping the TME. Notably, these macrophages engage in intense cross‐talk with both tumour cells and fibroblasts, reinforcing a tumour‐promoting network. This interaction between SPP1^+^ TAMs and fibroblasts appears to be a shared mechanism across multiple cancer types, where fibroblast‐macrophage communication fosters an environment conducive to tumour progression.

Despite these insights, many questions remain regarding the precise role of SPP1^+^ TAMs in HNSCC, particularly in relation to their plasticity, origin, and potential as therapeutic targets. However, their immunosuppressive, pro‐angiogenic, and ECM‐remodelling functions, along with emerging evidence of hybrid or non‐canonical phenotypes, underscore their adaptability and influence in supporting tumour progression.

### 4.5 Liver Cancer

The enrichment of SPP1^+^ TAMs in liver cancer mirrors their prominence in other malignancies, yet their role in this context appears to be particularly intricate. Not only are these macrophages present in greater numbers, but they are also closely associated with cancer stem cells, suggesting a role in sustaining tumour growth and progression [61]. Functionally, SPP1^+^ TAMs exhibit a transcriptional profile enriched in cytokines, chemokines, and metalloproteases, indicating a potent capacity for ECM remodelling. Spatial transcriptomics analyses have revealed that these macrophages may contribute to the formation of immunosuppressive barriers within the tumour microenvironment, potentially restricting immune cell infiltration and facilitating tumour immune evasion (Figure 2B). Similar findings from earlier studies have linked SPP1^+^ TAMs to high expression of *MMP9*, a key enzyme involved in matrix degradation and tissue remodelling [62].

A particularly intriguing aspect of SPP1^+^ TAMs in liver cancer is their terminally differentiated state and their dependency on PPARγ, a transcription factor known for its role in M2‐like macrophage polarisation and adipogenesis. PPARγ activation in these macrophages has been directly linked to their ability to promote hepatocellular carcinoma (HCC) cell migration, invasion, and tumour angiogenesis [62]. Interestingly, some reports suggest that SPP1^+^ macrophages display a foamy‐like morphology with enhanced lipid metabolism and phagocytic activity in atherosclerosis settings [63], raising the question of whether PPARγ‐driven metabolic adaptations contribute to their foamy‐like appearance, enhanced lipid metabolism, phagocytic activity, as well as tumour‐supporting functions in cancer contexts. This remains an exciting avenue for further investigation.

However, although SPP1^+^ TAMs have been generally correlated to a poorer prognosis and higher aggressiveness in many tumours [43, 64, 65, 66], different behaviours from this macrophage population have been observed in the context of liver cancer, possibly indicating specific, yet uncovered functions of SPP1^+^ TAMs. Indeed, a study on intrahepatic cholangiocarcinoma (ICC) revealed *S100P* and *SPP1* as two potential biomarkers to study this tumour heterogeneity, with SPP1^+^ TAMs numerosity being particularly high in ICC peripheral small duct type compared to ICC perihilar large duct type [67].

In liver cancer, SPP1^+^ TAMs emerge as highly specialised components of the tumour microenvironment, shaped by metabolic cues, spatial positioning, and possibly their developmental trajectory. Their association with PPARγ signalling and lipid‐rich, foamy‐like features points to a unique adaptation that may distinguish them from SPP1^+^ macrophages in other tissues. Rather than acting as a uniform pro‐tumour entity, these cells display context‐dependent behaviours, suggesting that their influence in hepatocellular carcinoma and cholangiocarcinoma is intricately tied to tumour subtype and microanatomy. Understanding these nuances will be key to deciphering their precise role in hepatic tumour biology and may open up new conceptual frameworks for studying macrophage diversity in cancer.

### 4.6 Lung Cancer

The pro‐fibrotic and tumour‐supporting roles of SPP1^+^ TAMs in lung cancer are becoming increasingly evident. Several studies, including data from the Human Lung Cell Atlas [68] single‐cell RNA sequencing on lung adenocarcinoma and squamous carcinoma [69], and multi‐omic profiling in non‐small cell lung cancer (NSCLC) [70], have identified a population of monocyte‐derived SPP1^+^ TAMs in lung cancer, reinforcing their significance in shaping the tumour microenvironment. This occurs through interactions with a plethora of different cell types, including tumoral cells, endothelial cells, and infiltrating immune cells. Additionally, another study on NSCLC has linked SPP1^+^ TAMs to pro‐angiogenic functions, further underscoring their role in tumour progression [71].

However, a key unresolved question is the functional heterogeneity of SPP1^+^ TAMs across different cancer types. While in liver cancer, PPARγ was identified as a TAM marker, the same transcription factor in lung cancer has been attributed to a distinct macrophage subpopulation. This discrepancy may stem from differences in analysis depth or tissue‐specific gene signatures, highlighting the plasticity of macrophage phenotypes in different tumours. Interestingly, PPARγ expression aligns with descriptions of SPP1^+^ macrophages as foamy cells, a feature reported in lung cancer TAMs expressing both *TREM2* and *SPP1*, where they have been shown to promote cholesterol efflux, fuelling tumour cell metabolism [72].

Further evidence of the tumour‐promoting role of SPP1^+^ TAMs comes from a murine lung adenocarcinoma model, where a population of TREM2+ macrophages, also expressing the SPP1^+^ gene signature (*TREM2*, *GPNMB*, *SPP1*, *CTSB*, *RNASE1*, *GPR183*), was identified [73]. These macrophages initially function as tumour debris scavengers (Figure 2C) but subsequently transition into a pro‐tumorigenic state, reinforcing the idea that macrophage function evolves in response to the tumour microenvironment.

Beyond their impact on tumour metabolism, SPP1^+^ TAMs have been shown to interact extensively with cancer‐associated fibroblasts (CAFs), a key process in tumour progression. A study on NSCLC identified SPP1^+^ TAMs engaging with COL11A1‐expressing CAFs at the tumour boundaries, promoting collagen deposition and extracellular matrix remodelling [70]. Additionally, computational analyses of cell–cell interactions across multiple studies have revealed enrichment of the SPP1‐CD44 signalling axis, which correlates with lower T‐cell infiltration and heightened immunosuppression [74].

In lung cancer, SPP1^+^ TAMs stand at the crossroads of fibrosis, metabolism, and immune regulation. Their dynamic interaction with cancer cells, fibroblasts, and the extracellular matrix suggests a multifaceted identity, one that evolves alongside the tumour. Rather than fitting neatly into established macrophage paradigms, these cells exhibit tissue‐specific adaptations, such as lipid‐handling capabilities and pro‐fibrotic behaviour, shaped by cues in the pulmonary microenvironment. Their transformation from debris‐clearing scavengers to metabolic and structural architects of the tumour landscape underscores their plasticity and influence. As we refine our understanding of this complexity, SPP1^+^ TAMs in lung cancer offer a compelling lens through which to study the interface between inflammation, fibrosis, and malignancy.

### 4.7 Other Cancers

The presence of SPP1^+^ TAMs has been reported across various cancer types, often exhibiting context‐dependent functional roles shaped by the tumour microenvironment. Despite this heterogeneity, SPP1^+^ TAMs consistently contribute to tumour progression through shared features such as immunosuppression, extracellular matrix remodelling, and metabolic reprogramming. In pancreatic ductal adenocarcinoma, a specific macrophage subpopulation expressing SPP1, along with *MARCO*, *FBP1*, *APOC1*, and *LIPA*, has been identified [75]. This expression profile suggests a role in lipid metabolism and phagocytosis, potentially shaping the metabolic landscape of the tumour microenvironment (Figure 2D). The foamy‐like morphology and lipid‐processing capabilities of these cells are features also observed in SPP1^+^ macrophage populations from other malignancies, highlighting lipid metabolism and phagocytic activity as hallmarks of this macrophage subclass.

Similarly, in clear cell renal cell carcinoma, SPP1^+^ TAMs exhibit an M2‐like signature, characterised by high expression of *CXCL9*, *CXCL10*, and the pan‐macrophage marker *CD68* [76]. This phenotype indicates an immunosuppressive and pro‐tumorigenic role, likely contributing to tumour progression and resistance to immune‐mediated clearance. The immunosuppressive role observed in clear cell renal cell carcinoma further supports the notion that SPP1^+^ TAMs may act as specialised effectors within the broader immunosuppressive TAM pool. However, the specific contributions of SPP1^+^ TAMs relative to other TAM subsets remain to be fully delineated, warranting comparative functional studies.

In bladder cancer, SPP1^+^ TAMs have been associated with increased angiogenesis and heightened activity in tryptophan metabolism, particularly through *IL4I1* [77]. These macrophages interact closely with regulatory T cells (Tregs), further modulating the immune response and reinforcing an immunosuppressive tumour microenvironment. A similar mechanism has been observed in oesophageal squamous cell carcinoma, where SPP1^+^ TAMs play a key role in recruiting CD4^+^ Tregs, leading to immune suppression and treatment resistance [78].

Table 2 summarises the common gene markers associated with SPP1+ TAMs discussed in this review.

**TABLE 2:** Markers of SPP1^+^ TAMs.

Marker	Tumour type	Model organism	References
APOE	Glioma	Mouse	[42]
APOC1	Glioma, Pancreatic cancer	Mouse, Human	[42, 43, 75]
LGALS3	Glioma	Mouse	[42]
GPNMB	Glioma, Lung cancer	Mouse, Human	[42, 73]
FTL	Glioma	Human	[43]
LAPTM5	Glioma	Human	
S100A11	Glioma	Human	
APOC2	Glioma	Human	
MARCO	Colorectal cancer, Pancreatic cancer	Human	[54, 56, 75]
FN1	Colorectal cancer	Human	[55]
CLEC5A	Colorectal cancer	Human	
OLR1	Colorectal cancer	Human	
IL1RN	Colorectal cancer	Human	
MT1G	Colorectal cancer	Human	[56]
CHI3L1	Colorectal cancer	Human	
NLRP3	Colorectal cancer	Mouse	[57]
TNF	Colorectal cancer	Mouse	
VEGFA	Colorectal cancer	Mouse	
ARG1	Colorectal cancer	Mouse	
MMP9	Liver cancer	Human	[62]
S100P	Liver cancer	Human	[67]
PPARγ	Liver cancer, Lung cancer	Human	[62, 72]
TREM2	Lung cancer	Human	[72, 73]
CTSB	Lung cancer	Human	[73]
RNASE1	Lung cancer	Human	
GPR183	Lung cancer	Human	
FBP1	Pancreatic cancer	Human	[75]
LIPA	Pancreatic cancer	Human	
CXCL9	Renal carcinoma	Human	[76]
CXCL10	Renal carcinoma	Human	
IL4I1	Bladder cancer	Human	[77]

*Note*: Overview of markers associated with SPP1^+^ tumour‐associated macrophages across different cancer types. The model organism in which each marker has been identified is indicated, along with the corresponding cancer type(s) and reference(s).

Collectively, while SPP+ TAMs demonstrate tissue‐ and cancer‐specific gene expression patterns and interactions, they share common traits such as enhanced lipid metabolism, immunosuppressive activity, and capacity to modulate T‐cell responses. These conserved functions, alongside their context‐specific behaviours, highlight SPP1^+^ TAMs as a distinct macrophage lineage with broad implications for cancer progression and therapeutic resistance. Understanding the shared and unique features of SPP1^+^ TAMs across malignancies may offer new opportunities for targeted interventions aimed at disrupting their tumour‐promoting roles.

## 5 SPP1 Macrophages in Non‐Cancerous Conditions

### 5.1 Skeletal Muscle

Macrophages play a fundamental role in skeletal muscle regeneration, orchestrating the transition from a pro‐inflammatory phase that recruits immune cells to an anti‐inflammatory phase that resolves inflammation and supports tissue repair [79]. In skeletal muscle, SPP1^+^ macrophages have been particularly studied in murine models of muscular dystrophies. They exhibit complex and sometimes opposing roles depending on the context (aging, disease, injury type), with evidence suggesting both beneficial and detrimental effects.

**FIGURE 3:**
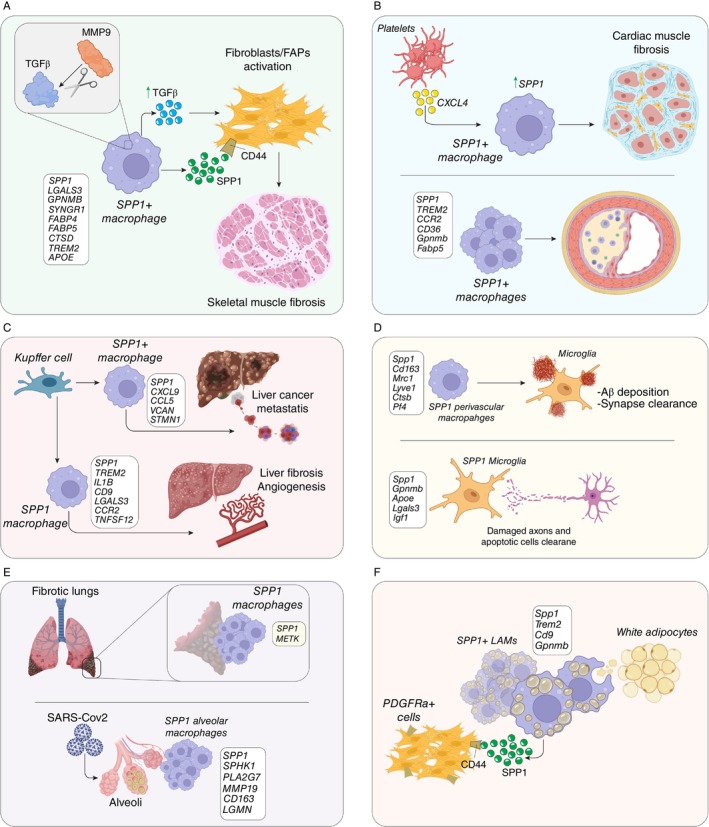
SPP1^+^ macrophages in tissues and non‐cancerous conditions. (A) In skeletal muscle pathologies, MMP9‐mediated TGF beta activation guides SPP1^+^ macrophages' interaction with fibroblasts to induce muscle fibrosis. (B) In the heart, platelet‐derived CXCL4 guides SPP1^+^ macrophages to induce tissue fibrosis. (C) In pathology, Kupffer cells‐derived macrophages expressing SPP1 enhance liver metastasis spreading, fibrosis, and angiogenesis. (D) In brain pathologies, an increment of SPP1^+^ macrophages leads to amyloid beta plaques deposition and damaged axons/synapses clearance. (E) In the fibrotic lung and SARS‐Cov2 infection, SPP1^+^ macrophages accumulate in the fibrotic regions and alveoli. (F) In the adipose tissue of obese patients, SPP1^+^ macrophages are responsible for the clearance of apoptotic adipocytes and interact with PDGFRα progenitors influencing the adipose tissue composition.

SPP1 has been detected in serum and biopsies from both human and mouse muscle [80], in calcified areas of mouse dystrophic muscles [81], and is strongly upregulated in dystrophic muscles, where it promotes fibrosis. This occurs partly by enhancing TGF‐β1 signalling through MMP9, a protease that releases latent TGF‐β1 from its inactive complex. Inhibition of MMP9 reduces collagen expression in dystrophic *mdx* fibroblasts [82] (Figure 3A). Indeed, in *mdx* models (a widely used mouse model for Duchenne muscular dystrophy), Spp1 ablation decreases active TGFβ and improves fibrosis, whereas in the mdxD2 model, a more severe dystrophic model characterised by constitutively high TGFβ signalling, Spp1 ablation has minimal impact. Interestingly, in juvenile mdxD2 mice, elevated Spp1 contributes to fibrosis, whereas in older mice, decreased Spp1 improves regenerative capacity by regulating macrophage polarisation [83]. Similarly, in aging muscle, a population of SPP1^+^ macrophages expressing *Gpnmb*, *Syngr1*, and *Lgals3* correlates with increased fibrosis, and Spp1 ablation enhances regeneration by shifting macrophages toward a pro‐regenerative phenotype [84, 85].

Some reports highlighted how SPP1^+^ macrophages accumulate in pathological muscle tissues and are generally associated with poor prognosis [86]. They accumulate in aged skeletal muscle and co‐express markers linked to senescence, aging, and lipid metabolism, such as *Fabp5*, *Ctsd*, *Trem2*, *Fabp4*, and *Apoe* [87]. In murine models of muscular dystrophy, these macrophages expand in response to tissue damage and, through autocrine and paracrine signalling, further promote fibrosis [58, 86]. Spp1 secreted by these macrophages interacts with fibro/adipogenic progenitors, promoting their differentiation into fibroblasts and increasing ECM deposition, which exacerbates fibrosis [88].

The mechanisms by which SPP1^+^ macrophages exert their functions are still under intensive investigation, and researchers are mainly focusing on the interactions between these macrophages and other tissue‐resident cell populations. Specifically, the SPP1–CD44 interaction has been identified as a key mechanism in aged skeletal muscle macrophages, activating fibroblasts and enhancing collagen production [89]. SPP1 can also induce LRRC15+ fibroblast activation, establishing a feedback loop that sustains macrophage polarisation and fibrosis [90].

Beyond skeletal muscle, SPP1^+^ macrophages play a role in degenerative ligament conditions by interacting with CRTAC1+ chondrocyte‐like cells, contributing to ligament degeneration [91]. Interestingly, in adhesive capsulitis (frozen shoulder), SPP1 is downregulated compared to non‐inflamed tissue, despite no significant changes in macrophage abundance [92], suggesting that the nature and chronicity of inflammation strongly influence the emergence and function of SPP1^+^ macrophages.

The story of SPP1^+^ macrophages in skeletal muscle is one of context and complexity. Rather than playing a fixed role, these cells shift identities based on age, disease state, and microenvironmental signals. They act as central players in the fibrotic response, not only by engaging fibroblasts through direct signalling pathways like SPP1–CD44, but also by creating feedback loops that reinforce tissue remodelling and chronic inflammation. At the crossroads of regeneration and degeneration, their influence extends beyond muscle into ligaments and joint tissues, adapting their function in response to inflammatory cues. This versatility makes them a powerful yet double‐edged force in musculoskeletal pathology—capable of driving repair or perpetuating damage, depending on the circumstances.

### 5.2 Heart

Cardiac fibrosis is a physiological response to injury that involves multiple cell types, including immune cells [93]. Macrophages play a central role in this process by orchestrating tissue regeneration and regulating mesenchymal cell activation [94]. Among the signals that activate macrophages, platelets appear to play a key role by secreting CXCL4, a chemokine that induces SPP1 expression in macrophages [58] (Figure 3B). Following heart injury, this SPP1^+^ macrophage population expands and interacts with fibroblasts to promote fibrosis. However, whether these macrophages represent a terminally differentiated population [95] or are instead stalled in an intermediate state [58] remains unclear and urges further investigation.

The pro‐fibrotic role of SPP1^+^ macrophages in the heart is particularly evident in models of heart failure, where they are enriched in perivascular adipose tissue surrounding atherosclerotic coronary arteries and are strongly associated with severe fibrosis [96]. In this study, as well as other studies on muscle tissue [88, 89, 91], cell–cell interaction predictions using single‐cell computational approaches suggest that SPP1^+^ macrophages communicate with mesenchymal cells via CD44, a well‐known SPP1 receptor. However, further experimental validation is required, as CD44 exists in multiple isoforms, not all of which contain the SPP1‐binding domain.

As observed in other tissues, SPP1^+^ macrophages in the heart exhibit enhanced phagocytic activity, at least at the transcriptional level. In the ischemic heart, these macrophages co‐express *TREM2*, *CCR2*, and *CD36*, with the latter being a key player in phagocytosis [97]. Furthermore, in a mouse model of myocardial infarction, SPP1^+^ macrophages have been shown to contribute to tissue remodelling and display high expression of *Trem2*, *Gpnmb*, and *Fabp5* [98].

While the precise mechanisms governing the role of SPP1^+^ macrophages in cardiac fibrosis remain an active area of study, their interactions with mesenchymal cells, such as fibroblasts, and their involvement in tissue remodelling after heart injury provide important clues to their functional significance. The complexity of their activation and differentiation, as well as their contribution to fibrosis through ECM modulation, underscores the need for further research to better understand their precise role in cardiac pathophysiology.

### 5.3 Liver

Kupffer cells (KCs), the resident liver macrophages, play essential roles in hepatic homeostasis, contributing to liver regeneration, immune tolerance, and host defence during both sterile and infectious challenges [99].

A cellular indexing of transcriptomes and epitopes by sequencing (CITE‐seq) analysis of metastatic liver nodules and adjacent normal tissue identified a population of SPP1^+^ macrophages. This subset represents a subpopulation of “lipid metastasis‐associated macrophages” (LMAMs), characterised by high expression of fibrosis‐related genes such as *Spp1* and *Trem2*, similar to scar‐associated macrophages described in liver fibrosis [100]. These findings suggest a potential role for SPP1^+^ macrophages in shaping the fibrotic and immunosuppressive microenvironment of liver metastases. In parallel, a distinct SPP1^+^ macrophage population has been identified in liver cirrhosis, characterised by the expression of *TREM2*, *IL1B*, *CD9*, *LGALS3*, *CCR2*, and *TNFSF12*. Unlike Kupffer cells, this subset, referred to as scar‐associated macrophages (SAMacs), has been implicated in promoting tissue fibrosis and angiogenesis [101] (Figure 3C).

SPP1‐secreting macrophages have also been identified in non‐alcoholic steatohepatitis (NASH) as a population distinct from Kupffer cells [102, 103]. However, studies report conflicting findings regarding the role of SPP1^+^ macrophages in liver disease. On one hand, macrophage‐derived SPP1 has been suggested to exert protective effects against liver steatosis by inducing arginase‐2 (ARG2), which enhances fatty acid oxidation in hepatocytes. On the other hand, increased numbers of SPP1^+^ macrophages in pathological conditions have led to their consideration as a potential biomarker of liver disease.

The functional diversity of these macrophages, as reflected by their expression of various markers such as *TREM2*, *IL1B*, and *CD9*, points to their complex roles in both promoting fibrosis and regulating metabolic processes. Further research is required to clarify the precise role of these macrophages in non‐cancerous liver conditions.

### 5.4 Brain and Central Nervous System

Microglia, the brain‐resident macrophages, play a fundamental role in maintaining central nervous system (CNS) homeostasis. SPP1 is expressed by microglia, as well as astrocytes, neurons, and oligodendrocytes, with its upregulation often associated with neurodegeneration and poor prognosis. In oligodendrocytes, SPP1 is linked to the myelination process, while in microglia, it plays a role in opsonization, phagocytosis, and proliferation. Astrocytic SPP1 expression has been correlated with astrogliosis and migration, whereas in neurons, it appears detrimental, as seen in iPSC‐derived neurons from frontotemporal dementia patients, where its expression is associated with mitochondrial dysfunction and gliosis [104, 105].

A recent CRISPR/Cas9‐based screening in iPSC‐derived microglia identified a population of SPP1^+^ microglia with enhanced phagocytic activity. The authors demonstrated that knockdown or pharmacological inhibition of MAPK14 or CSF1R modulates this disease‐associated microglial subpopulation, either promoting or depleting the SPP1^+^ state, respectively [106]. These findings may have important implications for brain disorders, as CSF1R inhibition has been shown to exert beneficial effects in mouse models of neurological diseases, including Alzheimer's disease [107], tauopathy [108], and multiple sclerosis [109].

In neurological disorders, the role of SPP1^+^ microglia is influenced by aging. In juvenile brain injury models, SPP1^+^ microglia, termed “damage‐associated microglia” (DAM), undergo cell death post‐recovery, while neonatal DAM exhibit plasticity, reverting to a homeostatic phenotype and showing immune memory [41]. In fetal development, SPP1^+^ microglia contribute to brain lesion repair and protection from morphogenetic stress and injury [110]. Additionally, SPP1 is predominantly expressed by perivascular macrophages, and in Alzheimer's disease models, its upregulation is linked to amyloid beta deposition and microglial synapse clearance, which can be either protective or detrimental depending on the disease stage [111] (Figure 3D). These SPP1^+^ macrophages shared markers with other tissue‐resident macrophages, including *Cd163*, *Mrc1*, *Lyve1*, *Ctsb*, and *Pf4*.

While SPP1 expression in microglia and perivascular macrophages is often associated with detrimental effects, some studies highlight protective roles in astrocytes. For example, SPP1 deficiency in murine astrocytes increases ganglion cell vulnerability to aging, intraocular pressure, and optic nerve damage, whereas its overexpression enhances visual function in glaucoma by promoting phagocytosis, neurotrophic factor secretion, mitochondrial function, and ATP production [112].

In a mouse model of thoracic spinal cord injury, a subset of activated microglia expressing *Spp1*—alongside markers such as *Gpnmb*, *Apoe*, *Lgals3*, and *Igf1*—was enriched in processes related to phagocytosis, lysosome function, and lipid metabolism, likely participating in the clearance of damaged axons and apoptotic cells [113]. Similarly, in ischemic stroke, SPP1^+^ myeloid cells accumulate near CD44+ oligodendrocyte progenitor cells (OPCs) and reactive astrocytes in the perilesional zone, promoting OPC migration and facilitating immuno‐glial cross talk [114].

It is evident that SPP1^+^ cells play critical roles in nervous system health and disease, with their effects dependent on spatial and temporal contexts. Further functional studies are necessary to clarify the roles of SPP1 and its expressing cells, potentially unlocking therapeutic opportunities targeting SPP1‐related pathways. Moreover, while studies on SPP1^+^ myeloid cells have shed light on their roles in the central nervous system, there remains a lack of evidence specifically addressing their function in the peripheral nervous system. This represents an important avenue for future research.

### 5.5 Lung and Respiratory Tract

One of the most comprehensive resources for lung cell data is the Human Lung Cell Atlas, which has identified a population of SPP1^+^ macrophages co‐expressing *LPL* and *CHIT1* across various disease conditions, including COVID‐19 and idiopathic pulmonary fibrosis (IPF). This work suggests a pro‐fibrotic role for these macrophages in the lung [68]. In support of this hypothesis, single‐cell RNA sequencing of IPF patients revealed an expansion of a highly proliferative macrophage population expressing *SPP1* and *METK*, particularly concentrated in the fibrotic lower lobes [115] (Figure 3E). Furthermore, spatial transcriptomics analysis confirmed the accumulation of SPP1^+^ macrophages in the airspaces of fibrotic lungs, distinguishing them from FABP4‐expressing macrophages, with evidence suggesting a differentiation process between these populations [116]. In murine models, a corresponding monocyte‐derived macrophage population (*SPP1* ^+^, *Gpnmb*+, *Fabp5+*, *Cd9+*, *Arg1+*) increased upon Notch2 downregulation, though without exacerbating fibrosis [117]. Notably, *SPP1* expression was also detected in pro‐fibrotic alveolar fibroblasts, indicating additional roles beyond macrophage‐driven fibrosis [118].

In the context of SARS‐CoV‐2 infection, a similar pro‐fibrotic monocyte‐derived alveolar macrophage (MoAM) population was identified, characterised by high *SPP1*, *SPHK1*, *PLA2G7*, and *MMP19* expression [119]. Notably, a comparable SPP1^+^ macrophage subset was observed in bronchoalveolar lavage fluid (BALF) samples from COVID‐19 patients, co‐expressing *CD163*, *SPP1*, and *LGMN*, and correlating with pulmonary fibrosis [120]. This suggests a substantial overlap between the MoAMs present in lung tissue and those recovered from BALF, indicating that both populations likely represent the same pro‐fibrotic macrophage state associated with severe COVID‐19.

Altogether, these findings underscore the central role of SPP1^+^ macrophages in orchestrating fibrotic and inflammatory responses across a range of lung diseases. Their consistent enrichment in fibrotic lesions, distinct transcriptional signatures, and spatial localisation within diseased lung tissue suggest that SPP1^+^ macrophages represent a conserved, pathogenic macrophage state that drives maladaptive tissue remodelling.

### 5.6 Other Diseases

SPP1^+^ macrophages have been implicated in a wide range of diseases beyond cancer, particularly those involving lipid metabolism and fibrosis. These macrophages, often referred to as lipid‐associated macrophages (LAMs) or foamy macrophages, have been extensively studied in disorders such as obesity [121] and inflammatory skin diseases [122]. *SPP1* itself serves as a key gene marker for lipid‐related conditions, with SPP1^+^ macrophages identified across diverse pathological settings, including adipose tissue dysfunction, liver disease, atherosclerosis, intestinal disorders, and cancer [123]. A defining feature of these macrophages is their co‐expression of *Cd9*, a marker commonly found in LAMs and scar‐associated macrophages (SAMs). Several studies suggest that SPP1^+^ macrophages, characterised by the expression of *Trem2*, *Cd9*, *Gpnmb*, and *Spp1*, play central roles in lipid‐related diseases [124], though their precise functions remain debated and sometimes contradictory.

In the context of adipogenesis, M2‐like macrophages expressing high levels of *SPP1* have been shown to clear dead white adipocytes, a process essential for maintaining tissue homeostasis. Interestingly, SPP1 acts as a chemoattractant for mesenchymal cells expressing *PDGFRα* and *CD44*, with interactions occurring via the SPP1‐CD44 axis [125] (Figure 3F).

However, the role of SPP1^+^ macrophages extends beyond adipose tissue. They have also been linked to bone resorption, where they activate osteoclasts to degrade the bone matrix [126]. In this setting, epididymal white adipose tissue emerges as a major source of SPP1, which in turn stimulates bone marrow‐derived macrophages to engulf lipid droplets released from bone marrow adipocytes, establishing a direct connection between lipid metabolism and bone degradation. Further supporting their involvement in bone remodelling, a SPP1^+^CD9^+^ macrophage population has been identified in rheumatoid arthritis, where these cells play a role in disease progression and potential remission mechanisms [127].

Table 3 summarises the common gene markers associated with SPP1+ macrophages across the different tissues discussed in this review.

**TABLE 3:** Markers of SPP1^+^ Macrophages.

Marker	Tissue type	Model organism	References
GPNMB	Skeletal muscle, Heart, CNS, Lung, Adipose tissue	Mouse	[84, 85, 98, 113, 117, 124]
SYNGR1	Skeletal muscle	Mouse	[84, 85]
LGALS3	Skeletal muscle, Liver, CNS	Mouse, Human	[87, 101, 113]
FABP4	Skeletal muscle	Mouse	[87]
FABP5	Skeletal muscle, Lung	Mouse	[87, 98, 117]
CTSD	Skeletal muscle	Mouse	[87]
TREM2	Skeletal muscle, Heart, Liver, Adipose tissue	Mouse, Human	[87, 97, 98, 100, 101, 124]
APOE	Skeletal muscle, CNS	Mouse	[87, 113]
CCR2	Heart	Human	[97]
CD36	Heart	Human	[97]
IL1B	Liver	Human	[101]
CD9	Liver, Lung, Adipose tissue, Bone	Human, Mouse	[101, 117, 124, 127]
CCR2	Liver	Human	[101]
TNFSF12	Liver	Human	[101]
CD163	Brain	Mouse	[111]
MRC1	Brain	Mouse	[111]
LYVE1	Brain	Mouse	[111]
CTSB	Brain	Mouse	[111]
PF4	Brain	Mouse	[111]
IGF1	CNS	Mouse	[113]
LPL	Lung	Human	[68]
CHIT1	Lung	Human	[68]
METK	Lung	Human	[115]
ARG1	Lung	Mouse	[117]
SPHK1	Lung	Human	[119]
PLA2G7	Lung	Human	[119]
MM19	Lung	Human	[119]
LGMN	Lung	Human	[120]
CD163	Lung	Human	[120]

*Note*: Overview of markers associated with SPP1^+^ macrophages across different tissues in disease contexts. The model organism in which each marker has been identified is indicated, along with the corresponding tissue type(s) and reference(s).

Overall, SPP1^+^ macrophages are implicated in a wide array of non‐cancerous diseases, particularly those involving lipid metabolism, fibrosis, and tissue remodelling. Their role extends beyond adipose tissue dysfunction and atherosclerosis, where they are central to processes like lipid clearance, tissue homeostasis, and osteoclast activation in bone resorption. In diseases such as rheumatoid arthritis and inflammatory skin disorders, these macrophages interact with key cellular players, including mesenchymal cells and osteoclasts, to modulate disease progression. The complex functions of SPP1^+^ macrophages, shaped by their marker expression and interactions within various tissue microenvironments, highlight their pivotal role in mediating both tissue repair and pathological changes across a range of diseases.

## 6 Concluding Remarks

SPP1^+^ macrophages emerge as a pivotal component of the tissue response to the local environment, both in cancerous and non‐cancerous diseases. While many aspects of their biology remain to be fully elucidated, certain defining features have become increasingly clear. Their role in shaping an immunosuppressive microenvironment stands out, as they exhibit a profile reminiscent of M2‐like macrophages while also displaying pro‐inflammatory traits. This hybrid state may contribute to their ability to modulate T cell infiltration, reinforcing their role in immune regulation.

Beyond immunosuppression, SPP1^+^ macrophages are deeply implicated in fibrosis, as evidenced by both their transcriptional profile and their interactions with stromal cells, whether cancer‐associated fibroblasts or fibroblast progenitor cells. This dual capacity to drive fibrosis and suppress immune responses suggests that they may serve as a critical link between these two pathological processes, with their impact varying depending on the tissue, disease type, and microenvironmental cues.

Their pro‐angiogenic properties add another layer to their functional significance, particularly in the context of cancer. This makes SPP1^+^ macrophages an attractive therapeutic target, with potential strategies aimed at disrupting their role in angiogenesis and immunosuppression while enhancing the efficacy of chemo‐ and immunotherapies. At the cellular level, these macrophages also display enhanced phagocytic activity and increased lipid metabolism, features that may be instrumental in extracellular matrix remodelling and shaping the local microenvironment.

Despite these insights, many questions remain. Single‐cell RNA sequencing studies have highlighted distinct transcriptional profiles for SPP1^+^ macrophages across different datasets, raising the need for functional validation and deeper characterisation. Potential biases, stemming from sequencing depth, platform variability, data analysis pipelines, and clustering methodologies, may influence the observed gene signatures [128]. Additionally, many reported cell–cell interactions rely on computational predictions, which require further experimental validation, a challenge that is particularly pronounced when working with human samples. Some studies leveraging spatial transcriptomics have begun addressing this gap, but much remains to be explored.

Another layer of complexity comes from the diverse isoforms of CD44, one of the best‐characterised receptors of SPP1. Not all CD44 isoforms possess a binding site for SPP1, suggesting that interactions involving SPP1^+^ macrophages may be more nuanced than currently appreciated. Moreover, while SPP1 is a defining feature of these macrophages, it is likely not the sole driver of their function. A network of secreted molecules and transcription factors may work in concert to determine their behaviour, emphasising the need to study SPP1^+^ macrophages as a population rather than focusing solely on SPP1 itself. This is further supported by the fact that SPP1 is also produced by non‐macrophage cells [129], exerting effects through both autocrine and paracrine signalling.

In cancer settings, while SPP1^+^ macrophages share core features across cancer types, including pro‐fibrotic and immunosuppressive functions, their behaviour in metastatic settings may diverge from that in primary tumours, warranting further investigation into the influence of the metastatic microenvironment.

As research continues to uncover the complexity of SPP1^+^ macrophages, integrating functional studies, spatial transcriptomics, and advanced in vivo models will be key to unlocking their full therapeutic potential. Understanding how they orchestrate immune suppression, fibrosis, and angiogenesis across diseases may open new avenues for targeted interventions, paving the way for innovative strategies in immunotherapy and fibrosis management.

## Conflicts of Interest

The author declares no conflicts of interest.

## Data Availability

Data sharing not applicable to this article as no datasets were generated or analysed during the current study.
